# The impact of COVID-19 outbreak and perceptions of people towards household waste management chain in Nepal

**DOI:** 10.1186/s40677-021-00188-w

**Published:** 2021-06-23

**Authors:** Ashis Acharya, Govinda Bastola, Bindu Modi, Asmi Marhatta, Suraj Belbase, Ganesh Lamichhane, Narayan Gyawali, Ranjan Kumar Dahal

**Affiliations:** 1 Central Department of Geology, Tribhuvan University, Kirtipur, Nepal; 2 Central Department of Statistics, Tribhuvan University, Kirtipur, Nepal; 3 Central Department of Chemistry, Tribhuvan University, Kirtipur, Nepal; 4 Rural Education and Environment Development Center, Lalitpur, Nepal

**Keywords:** COVID-19, Household waste, Satisfaction level, Waste policy

## Abstract

The spread of COVID-19 is posing significant challenges to the household (HH) waste management sectors putting waste personnel and concerned bodies under massive pressure. The chain of collection, segregation, recycling, and disposal of household generated wastes is interrupted. This study aimed to assess how the household waste management chain was disrupted by novel coronavirus in Nepal and find the perception of the people towards the existing household waste management system (HHWMS). A descriptive online survey was carried out among 512 people using a cross-sectional research design and data was collected through a self-administered questionnaire method. Both descriptive, as well as inferential tests, were conducted using SPSS software. The finding of this study showed that 62.3% of respondents were not satisfied with the present HHWMS. Furthermore, there was a significant association of the satisfaction level of household waste management during coronavirus outbreak with gender, waste volume change in lockdown, PPE for waste collectors, and education on waste handling techniques provided by the government sector at 5% level of significance (*p* < 0.05). Proper HH waste management has become a challenge, and to address this some innovative works such as awareness programs for people, health and hygiene related support to waste workers, and effective policy formulation and implementation should be done by the Government of Nepal.

## Introduction

The present COVID-19 disease initially named 2019-nCOV, is known as severe acute respiratory syndrome coronavirus-2 (SARS-CoV-2) which is similar to two previous outbreaks, severe acute respiratory syndrome coronavirus (SARS-CoV) and middle east respiratory syndrome coronavirus (MERS-CoV) (Perlman 2020). The COVID-19 pandemic portrays this era’s major global public health concern as it has taken the life of 2.45 million; however, 62.3 million people recovered defeating the Covid-19 (Coronavirus (COVID-19) 2021). In total 111 million, people are affected. COVID-19 is extremely infectious and has spread worldwide swiftly; transmission from individual to individual happens mainly by direct contact or via droplets released by an infected person through coughing or sneezing. In certain cases, though, it seems asymptomatic (Rothan and Byrareddy 2020)**.** The virus was first observed in people, who were exposed to a seafood market in Wuhan, China. In Nepal, a 32-year-old Nepalese student from Wuhan, China, was found positive for the 2019-nCoV real-time PCR test on January 13, 2020, and Nepal went into nationwide lockdown. Due to lockdown along with other sectors, particularly the globe faced the major problem in solid waste management (SWM). Consequently, the waste collection centers became almost functionless and severely impacted (Mihai 2020; Sarkodie and Owusu 2020). Similarly, the impact extended to the waste-to-energy (WTE) and waste-to-material (WTM) industries (Zhou et al. 2021). The outbreak has added extra sources of waste products which have ultimately triggered even more complexity in the management of SW to governments and waste workers (Sharma et al. 2020a, 2020b; Tripathi et al. 2020).

One of the common sources of waste is plastics and its reduction is a global concern, and to minimize plastic production and use, globally plastic reduction policy is being formulated and implemented; however, the plastic reduction policy has also been severely disrupted which increased plastic waste volume (Patrício Silva et al. 2020). The COVID-19 crisis has unleashed a plastic disaster, reversing the achievement of a decade of activism against single-use plastic worldwide, including in Nepal (Awale and Kumar 2020). The sudden intensification of single-use products (masks and gloves) and panic buying is reported because of the fear of the virus which has increased plastic pollution in the environment (Patrício Silva et al. 2021). It is reported that the monthly estimated use of 129 billion face masks and 65 billion gloves globally, is resulting in widespread environmental contamination (Vanapalli et al. 2021). As COVID-19 spreads to the developing world, with restricted access to medical support, expanded numbers of cases are doing self-care at home which is considered as a major source of the waste contaminated with the virus and its poor management threats to waste operators and others of the spread of the COVID-19 infection (Environment 2020).

As a consequence of the pandemic, the volume and sources of waste generation change; however, the traditional causes of environmental pollution from the different sectors like transportation, aviation, and industries have declined sharply (Myllyvirta 2020; Sarkodie and Owusu 2020). Similarly, the food production and consumption system has undergone significant changes and ultimately resulted in the sudden and radical change in the daily life of people worldwide and resulted in adverse effects on lifestyle, eating habits, and other household characteristics (Cosgrove et al. 2021; Di Renzo et al. 2020; Principato et al. 2020; Scacchi et al. 2021).

Due to fear of being infected by the virus, human’s new lifestyles like staying at home, restricted travel, online shopping and stockpiling foods, etc. have contributed tremendously to household wastes (Benker 2021; Hao et al. 2020). However, only a few studies have been reported on household waste management during COVID-19. Di Renzo *et. al.* revealed that household food loss and waste (FLW) has increased by 12% from extra-domestic consumption during pandemics (Aldaco et al. 2020). In Canada, the short-term changes in household waste flow due to COVID-19 disruptions resulted in changes in garbage production, reuse, and reduction practices, and changes in waste diversion and education have been reported (Ikiz et al. 2021). Likewise, a survey conducted in Morocco depicted that 87% of respondents mix coronavirus protective equipment with household waste, which may contribute to the spread of the virus (Ouhsine et al. 2020)**.**

In recent days, many researchers have been working to explore whether COVID-19 spreads from waste materials or not. Patients infected by the virus being treated at home are creating tainted waste conceivably disposed of as domestic waste (Mol and Caldas 2020), which can pose risks to waste laborers and employers (United States Department of Labor 2020). Likewise, for the first time, Yuan et al. pointed to the possibility that SARS-CoV-2 might spread by sewage (Yuan et al. 2020). Coronavirus may survive for hours to days in metals, cardboard, plastic, when these materials are arbitrarily dumped, they may jeopardize the lives of waste management workers. The scenario becomes much more serious for waste management workers in developing countries that are not capable of providing enough safety measures and hygiene practice, for instance, adequate personal protective equipment (PPE). The probability of the speed-up of COVID-19 is very high in ASEAN (Association of Southeast Asian Nations) countries where the waste management system is very weak (Kojima et al. 2020). Consequently, garbage collectors of those countries are at a high risk of being contaminated by a virus (Kampf et al. 2020). Moreover, novel research on COVID-19 indicates that the virus infects the human GI tract and is excreted into sewage which imposes a risk of waterborne transmission of the coronavirus (Carbone 2020). In the context of Nepal, the present pandemic led to an abrupt change in the waste management chain and, because of the lack of safety means, there is a high risk of exposure of waste workers and waste pickers towards coronavirus.

In Nepal, being an economically less developed country, there are very few studies about the impact of novel coronavirus on the waste management sector to date though it is one of the signing members of the Paris agreement. For this study, a survey has been carried out among 512 respondents all over Nepal via an online google form to delineate the existing situation and perception of the people towards the household waste management system (HHWMS). Additionally, this research article attempts to summarize the policy gaps in solid waste governance in the context of Nepal and the linkages of the impact in SWM amidst the Covid-19 pandemic.

### Current situation of COVID-19

**Fig. 1:**
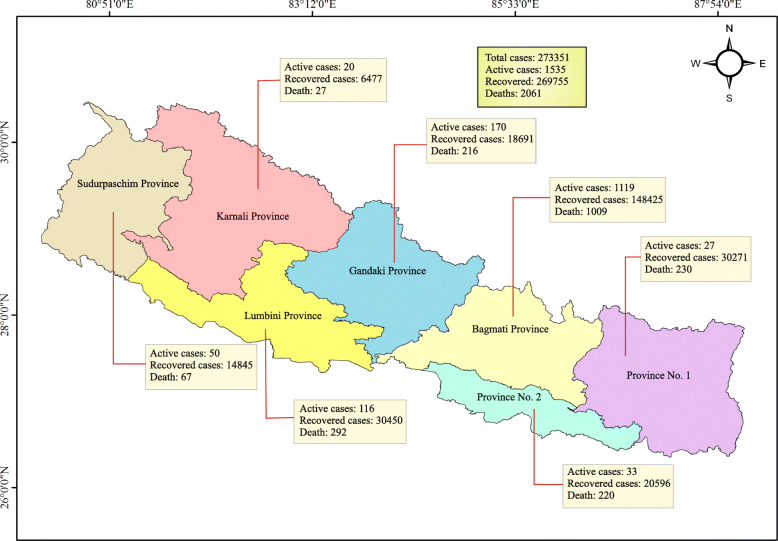
Map of Nepal depicting the present scenario of COVID-19 in all seven provinces

By February 20, 2021, 273 K total cases along with 2061 deaths and 270 K cases have been reported in Nepal (Fig. 1). Among the tested population, the case-fatality rate was 0.8%, and 98.7% recovery rate (CoVid19-Dashboard: MoHP 2021). Initially, as it was believed that the lockdown was supposed to be an influential method in reducing coronavirus spread throughout the world (Poudel and Subedi 2020), the national lockdown was imposed in March. Presently, the infection rate has fallen steadily, from an average of 3000 daily cases in October 2020 to about 300 in January 2021 (CoVid19-Dashboard: MoHP 2021), however, experts are claiming that there has been limited testing and warn that the country is still vulnerable to new strains. Although the number of cases going down, Nepal has implemented different precautions such as social distancing and hygiene practices to prevent COVID-19 under the governance of the Ministry of Health and Population. COVID-19 and other epidemic situations can be Nepal’s learning experience not only on evacuation and response but also on establishing a robust monitoring mechanism and implementing proactive measures in the possibility of similar incidents in near future (Shrestha et al. 2020). The present health care system is still not satisfactory. Indeed, we need proper practice and sufficient funding for the advancement of contact tracking tools, healthcare facilities, boosting public health services by voluntary cooperation, and greater use of mass media to mitigate the effect of the COVID-19 outbreak (Sapkota et al. 2020).

### Overview of solid waste management practices in Nepal before and during COVID-19

**Fig. 2:**
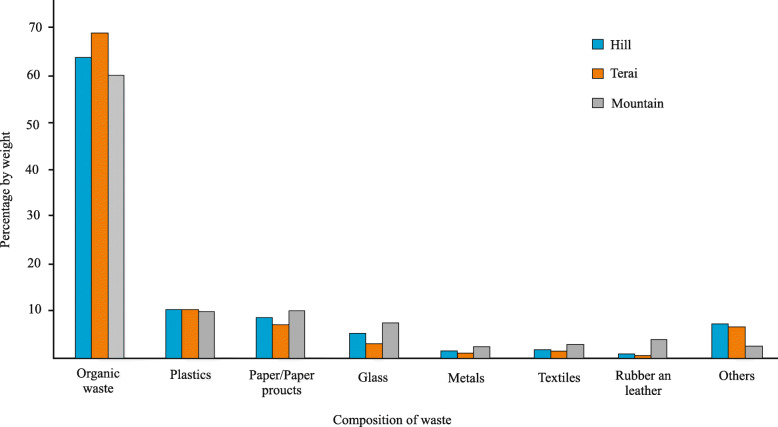
Average composition of waste in different ecological regions (after Pathak et al. 2020)

In developing countries, particularly in urban areas, the field of SWM is a highly neglected area (Masood et al. 2014; Narayana 2009; Ngoc and Schnitzer 2009; Shekdar 2009) and Nepal could not be the exception for this problem. There are only a few studies conducted to collect the SWM baseline information of Nepal (Alam et al. 2008; Asian Development Bank 2013; Dangi et al. 2009, 2011) but they are limited to old municipalities’ frameworks. With time, the population rose, and then “The 2011 Solid waste management act” was passed by the Government of Nepal. The act seeks to uphold a sustainable and stable atmosphere by reducing the detrimental impact of solid waste on public health and the ecosystem (Asian Development Bank 2013). Recently, Pathak et al. conducted an SWM baseline study to quantify MSW and its composition and to compile reliable information on the state of MSWM in the 60 newly formed municipalities of Nepal (Pathak et al. 2020). The average composition of waste in different ecological regions is varied from organic wastes, plastics, paper-products, glasses, metals, textiles, rubber-leather, and other different compositions. According to Fig. 2, the amount of organic wastes materials is high compared to other waste materials in all regions of Nepal. Regarding the number of paper-products, metals, textiles, and rubbers, this composition is in high proportion in the mountainous region whereas the Terai region ship the lesser amount. However, the area of SWM in Nepal is highly neglected and it has been challenged for over a decade concerning a proper recyclable and fruitful landfill site (Pathak et al. 2020). Only six municipalities in Nepal, namely Kathmandu, Lalitpur, Pokhara, Dhankuta, Tansen and Ghorahi practice open dumping of sanitary landfills for waste management and other municipalities have not practiced yet which has resulted from major environmental and human health risks (Dahal and Adhikari 2018). Moreover, unnecessary waste disposal is seen near water resources such as the banks of the river, the highways which led to water contamination along with waterlogging and drainage blockage, triggering hazardous health and environmental problems.

The COVID-19 crisis has had tremendous impacts on the waste management chain especially in those countries having low financial and technical capability as the lockdown or other restrictive measures are progressively lifted (Nzediegwu and Chang 2020; Shammi and Tareq 2020). In several countries, surveys are conducted to assess the situation and provide a summary and statistics on waste collection and treatment activities during the lockdowns (Belhadi et al. 2020; Zand and Heir 2020). But Nepal is lagging in such kinds of studies which depicts that Nepal has not been able to give priority to the field of waste management. One study mentioned that healthcare institutions have been facing various challenges as many types of infected waste are generated with a large volume of non-infected waste (Sharma et al. 2020a). The amount of contaminated waste has been increasing exponentially. The dispersion of abandoned masks and gloves outside indoor environments is creating environmental problems. At the household level, COVID-19 related wastes are not separated while disposed and mixed with Waste segregation and recycling have been limited to pilot projects and garbage trucks face problems in transporting waste to the landfill sites (Himalayan Times 2020). Currently, the Nepal government has assigned the “The solid waste management (SWM) act 2011” for the management of solid wastes. However, Nepal could not establish new guidelines related to solid waste collection, delivery, withdrawal, transport, treatment, and disposal during the mode of the pandemic.

## Materials and method

### Sampling and survey design

An online survey was conducted via google form using well-structured questionnaires to know the current situation of household waste management systems. This study covered a sample size of 512 using simple random sampling which truly represents the nation-wide population so that the finding of this study may be generalized. The survey was conducted in the period from 22 August until 5 September 2020 for 15 days.

### Data collection tools and techniques

All the information was compiled by conducting an online survey (self-completion questionnaire method), collecting relevant information from published and unpublished research (Nature, MDPI, Science News, SAGE, Lancet Public Health, and Preprint), and reports related to solid waste management in Nepal.

### Statistical analysis

Data obtained from the survey were analyzed using MS Excel and SPSS software. The cross-sectional study of this research involved both descriptive and inferential methods. The descriptive analysis consisted of frequency, percentage, cumulative percentage, etc. The Cross-Tabulation technique was employed to display a breakdown of the data. A chi-square test for independence also called Pearson’s chi-square test of association was used to determine whether there was a significant relationship between two categorical variables (Franke et al. 2012; Rana 2015). The chi-square test was calculated by using the formula,

$$ {\left(\chi \right)}^n=\sum \left(\frac{{\left({O}_i-{E}_i\right)}^2}{E_i\frac{\ }{\ }}\right) $$

With a degree of freedom (DF) = (r-1) × (c-1), where c denotes the number of levels for one categorical variable, and r denotes the number of levels for the other categorical variable. Likewise, *O*_*i*_ represents observed frequency and *E*_*i*_ represents the expected frequency under the null hypothesis which was computed separately for each level of one categorical variable at each level of the other categorical variable. Based on the test statistics and the DF, the *P*-values were also determined at a 0.05 level of significance.

## Results and discussion

### Demographic profile of respondents

The total number of respondents in this survey was 512. Out of this, 66.2% of the respondents were female and 33.8% were male. For the analysis, the data has been categorized into the following groups: 15–30 years, 30–50 years, and above 50 years. Of the total samples, it was observed that 92.6% of the respondents belong to the age group of 15–30. The percentage of respondents belonging to the age group of 30–50 is 6.8% and only 0.6% of respondents fall in the age group of above 50. The result further illustrated that more than half of the respondents (54.1%) were from the Bachelor degree’s level and 37.7% were from the Master degree’s level of education. Likewise, only 8.2% of respondents belonged to the high school level.

### Descriptive analysis

#### Dependent variable

The outcome variables of the study that is the satisfaction level of people on household waste management were categorized into two groups: satisfied and not satisfied. Participants were asked a question about their satisfaction with the present status of HH waste management. The results highlight that most of the respondents (62.3%) were found to be dissatisfied with the present waste management approaches while the rest of the respondents were found to be satisfied.

#### Independent variable

The statistical result of survey data showed that ¾^th^ (76%) of total respondents had fear of COVID-19 while the rest are not afraid of infection. It was also observed that almost 40% of people have been quarantined at their homes. Due to the outbreak of COVID-19 and some restrictive measures imposed by the government, 34.8% of people thought that the generation of household waste during lockdown and curfew had been increased and 57.8% of respondents believed that it remained constant. But very few (7.4%) people believed the decrease of the waste volume. Likewise, 56.3% of people always separated household waste at their homes before sending it to the recycling stations or disposal sites, and 34.2% of people separated waste sometimes. In contrast, the data also showed that there were still some people (9.6%) who never separated their household waste at the source level. Likewise, nearly two-thirds of respondents believed that the separation of waste at the source was not a challenging task.

The data further depicts that 54.7% and 22.5% of people have been getting the collection service from the government and private sector respectively. Unfortunately, it is seen that 22.9% of people did not have any access to waste collection services. In those areas where waste collection services are available, nearly 2/3^rd^ waste pickers do not wear PPE. Similarly, it was also observed that almost 70% of total respondents put garbage in nearby containers and nearly 20% of respondents burn their household waste. But the remaining 10% of people disposed of waste materials in open spaces, roads, water bodies, etc. During the pandemic, nearly 87% of people did not get any formal education by the government on household waste management techniques whereas 13% of people were found to be educated by the government on the waste handling process. Nearly 61% of people responded that the waste produced from houses should be transferred into recycling stations and others responded that it might be harmful and impose the risk of transfer of viruses if we sent household wastes into recycling centers.

#### Bivariate statistics

In this study, the bivariate analysis was adopted comparing the association between satisfaction levels of people regarding household waste management approaches during the lockdown and different categorical variables (Table 1).

**Table 1:** Satisfaction level of people concerning different categorical variables

Variables	Categories	Satisfaction level		Chi-square (p-value)
		Not satisfied	Satisfied	
		Number (percent)	Number (percent)	
Gender	Male	127 (73.4)	46 (26.6)	13.721 (0.000)
	Female	192 (56.6)	147 (43.4)	
Education level	High School	21 (50)	21 (50)	5.129 (0.077)
	Bachelor’s degree	168 (60.6)	109 (39.4)	
	Master’s degree and above	130 (67.4)	63 (32.6)	
Fear of COVID-19	Yes	251 (64.5)	138 (35.5)	3.397 (0.065)
	No	68 (55.3)	55 (44.7)	
Family in quarantine	Yes	132 (65.3)	70 (34.7)	1.314 (0.252)
	No	187 (60.3)	123 (39.7)	
Waste volume change during the lockdown	Increase	122 (68.5)	56 (31.5)	6.143 (0.046)
	Decrease	26 (68.4)	12 (31.6)	
	Constant	171 (57.8)	125 (42.2)	
Separation of waste at source	Always	178 (61.8)	110 (38.2)	0.219 (0.896)
	Sometime	109 (62.3)	66 (37.7)	
	Never	32 (65.3)	17 (34.7)	
Challenges of separating waste at source	Enough	36 (65.5)	19 (34.5)	1.789 (0.617)
	For nothing	15 (75)	5 (25)	
	Much	42 (61.8)	26 (38.2)	
	Not much	226 (61.2)	143 (38.8)	
Collection service	Government	161 (57.5)	119 (42.5)	6.883 (0.032)
	Private	75 (65.2)	40 (34.8)	
	No service	83 (70.9)	34 (29.1)	
PPE for waste workers (where collection service is available)	Yes	48 (40.7)	70 (59.3)	32.379 (0.000)
	No	271 (68.78)	123 (31.21)	
Transformation of covid-19 from wastes	Yes	194 (66.4)	98 (33.6)	5.684 (0.058)
	No	33 (52.4)	30 (46.6)	
	May be	92 (58.6)	65 (41.4)	
Dumping location	Burn	69 (66.3)	35 (33.7)	8.772 (0.067)
	By the road/street	8 (88.9)	1 (11.1)	
	Rivers/lakes	11 (78.6)	3 (21.4)	
	Nearby container	210 (58.8)	147 (41.2)	
	Open spaces	21 (75)	7 (25)	
Education by government authority about the waste management	Yes	27 (40.9)	39 (59.1)	14.768 (0.000)
	No	292 (65.5)	154 (34.5)	
Agree with recycling during a pandemic	Yes	194 (62.2)	118 (37.8)	3.109 (0.211)
	No	54 (70.1)	23 (29.9)	
	May be	71 (57.7)	52 (42.3)	

(at 5% level of significance)

From the above table, it is seen that the satisfaction level of waste management in the lockdown period among females was better as compared to that of male respondents. It means if we look at the total number of male and females separately, there were many people who were dissatisfied with peasant waste management. The p-value is less than 0.05, which indicates that there is a significant association between the satisfaction level of household waste management during a lockdown of the people and gender at 5% a level of significance. Similarly, waste volume changes during pandemic crisis have also a significant association with satisfaction levels of HH waste management. Similarly, PPE for waste collectors and education on waste handling techniques provided by the government sector has a significant association with present HH waste management approaches at 5% level of significance (*p* < 0.05). It means each variable directly affected HH waste management systems and this is supported by other studies (Kharel 2020).

In contrast, the bivariate statistical analysis highlighted that the education level of respondents was insignificant with waste management at 5% level of significance (*p* = 0.077). It portrays those respondents who have higher education levels are dissatisfied with the current waste management approach implemented by concerned bodies. Likewise, family in quarantine, fear of COVID-19 infection, separation of wastes at the source level, perception whether spread from waste materials, challenges of separation of wastes, location of disposing of generated wastes, and agree with waste recycling during pandemic have no any significant association with HH waste management approach at 5% level of significance.

### Challenges of HH waste management amidst COVID-19

Household waste, agriculture waste, industrial waste, hotel and, grocery waste has been the major source of the waste; however, household waste is a major contributor to municipal solid waste in developing countries like Nepal. It is estimated that the waste generated from the household accounts for about 65%- 70% of total municipal solid waste in Nepal (Pathak et al. 2020). Furthermore, this study found that the amount of household waste has been increased during this pandemic because of medical masks, tissues, and gloves. In turn, this excessive use of masks and hand gloves has increased the amount of household waste. Due to lack of resources such as staff, the door-to-door collection of such wastes is a major challenging issue. The study has found that there is a lack of waste segregation at the source due to the reason that the waste collectors mix all segregated wastes in the same dumping truck. Also, respondents from major cities responded that segregation of waste in separate bins is difficult due to limited space available at their homes. Public apathy due to the lack of awareness in in-situ segregation of wastes such as clinical and other kinds of daily-generated wastes at the household level is considered highly contagious, for instance, a similar case was observed in Iran (Zand and Heir 2020).

During the pandemic, waste collectors need to take additional precautionary measures while handling wastes. Household clinical waste generated during the pandemic can be highly contagious; therefore, collectors without a proper protective suite are highly prone to infection. In the nutshell, collectors/waste workers due to lack of PPEs facility, manual sorting of waste, and awareness on proper wearing of PPEs while handling HH wastes and waste from quarantine stations is being an alarming challenge to prevent from exposure of virus. The consequences of this could lead to a serious problem in the waste collection sector. Initially, the new Coronavirus is thought to be transmitted through person to person contact, however, a recent study published in the New England Journal of Medicine has found that the virus that causes COVID-19 could remain for an hour up to two to three days on the surfaces of the various type of contaminated materials and for several hours in the aerosols (van Doremalen et al. 2020).

The COVID-19 crisis also has posed a set of challenges to the policy makers arrive at a decision to ensure sustainable waste management (Ganguly and Chakraborty 2021). In Nepal, a recent waste management system entails dumping both at open places or improperly constructed disposal sites which that are not environmentally friendly. Owing to the shortage of human and financial resources leads to a difficult challenge for disposal of waste even in a minimum sanitary condition (Shakya and Tuladhar 2014). Thus, solid waste management is emerging a critical concern for municipal authorities especially in developing countries like Nepal due to the rapid surge in the quantities of MSW generated due to population growth in metropolitan areas, inadequate facilities like vehicles and transportation, and poor road infrastructure (Acharya 2016). Additionally, political turmoil, malfunctioning local authorities, and inadequate MSW treatment facilities have raised difficulties in the waste management system (Rai et al. 2019). For an instance, in case of the Sri-Lanka, despite the Local Governments bodies and other national government agencies have initiated numerous projects in coordination with concerned waste management authorities, most of them have not been effective due to inadequate land for disposal sites, composting and recycling, an advanced SWM network, lack of the appropriate equipment and modern technologies (Fernando 2019).

### Policy gaps for household solid waste governance in Nepal

In Nepal, solid waste management is a major challenge for urban and suburban areas where the number of immigrants has been rapidly increasing. The quarantine regimes have driven people to online shopping of daily utilities such as food and groceries. As a result, household-generated inorganic waste, as well as organic waste has escalated (Zambrano-Monserrate et al. 2020); however, in case of Nepal, the local government is unable to apply innovative approaches for the management of solid waste. During such crises, inadequate management of municipal waste management poses potential risks which may magnify the rapid spread of the virus among humans (Kulkarni and Anantharama 2020). Therefore, the codification of new policies regarding municipal waste management or their revision is necessary during the pandemic (Oyedotun et al. 2020; Torkashvand et al. 2021). Government of Nepal has several laws, including the Management and Resource Utilization Act 1987, the Labor Act 1991, the Municipal Act 1992, the Industrial Enterprise Act 1992, the Environmental Policy and Action Plan 1993, the Solid Waste Management National Policy 1996, the Environmental Protection Act and Rules 1997, the Local Self-Governance Act 1999. But, the implementation of laws of waste management systems are indeed in an incipient stage (Dangi et al. 2017).

To minimize the spread of the virus from wastes during the COVID-19 crisis, many countries have established new guidelines for SWM (Di Maria et al. 2020). In the case of Nepal, Solid waste management law, 2070 (2013), a legislative requirement of Nepal’s Solid Waste Management Act and Regulation, was assigned as a guideline but has not been properly applied and monitored. The Nepal government has also developed a second nationally determined contribution document (NDC) which has prioritized focusing on management of the municipal waste and committed to adopt and implement waste segregation, recycling and WTE programs in at least 100 municipalities by 2030; however, it does not sufficiently articulate the way it addresses the issue raised in the case of pandemic. In particular, MSW’s collection, sorting, distribution, and final storage is the responsibility of the local government. Due to the lack of finance and human resources, the local authorities provide only limited service to civil people (Kharel 2020). Metropolitan areas in Nepal are no edge cases; both local, as well as national authorities are experiencing major waste management problems. However, solid waste management isn’t even a primary concern for local municipalities as demand for many other public services in many municipalities in Nepal.

### Further recommendations

In Nepal, people who have tested positive for COVID-19 and have mild or no symptoms are living in home isolation according to the WHO protocol. Though the government of Nepal has strictly tried to implement WHO protocol at home (WHO 2020), the municipalities have not been able to collect and separate the infectious waste from home isolation. Infectious and non-infectious waste is collected and disposed of in the same place which is creating a frightening situation. Because of the unpleasant situation, infectious garbage coming out of the house quarantine should be well packed in a tight bag and closed completely before disposal and eventual collection by municipal waste services. In the absence of such facilities, the waste should be safely buried in a pit or incinerated without affecting the environment. Furthermore, arrangements should be made to provide necessary training on waste collection, sorting and transportation to the employees who are continuously working in waste management (Thompson 2020). Waste collectors are also one of the front-line workers during the pandemic. International solid waste association (ISWA) suggests that all governments should recognize the key-role of waste workers and the waste sector this period (Ganguly and Chakraborty 2021; Scheinberg et al. 2020). The government should provide insurance cover and other socio-economic support to them. For instance, in the United Kingdom (UK), the workers involved in sanitization have been given the status of ‘Key Worker’ in order to ensure safe schooling and care provision for their children and families by the government (Waste Disposal Included on “key Workers” List 2021). Likewise, Sharma et al. focused on the need for building localized resilient supply chains to counter such situations during future pandemics (Sharma et al. 2020a, 2020b). For underdeveloped countries in Africa, Belhadi et al. recommended a combined incineration and chemical disinfection approach, and combined chlorination and ultraviolet irradiation as the most sustainable technologies for managing infectious wastes (Belhadi et al. 2020). In developing countries like Nepal, landfill sites are not technically viable and environmentally friendly. Therefore, several other simple means of non-hazardous waste remediation such as composting, vermicomposting etc., should be undertaken to manage the common HH waste in an integrated manner.

In addition, each municipality/local authority has to develop contingency plans that ensure essential waste services remain uninterrupted and minimize the health risks. All soiled or infected materials should be collected and ideally segregated as per biomedical waste type, and thereafter placed into clearly labeled leakproof plastic bags (appropriately colored where possible) or designated containers (e.g. puncture-proof sharp boxes). In Wuhan, China, internet of things (IoT) technology was employed for the disposal of waste which made a real-time tracking and controlling process. Such automatic processes use a minimum number of workers and include sensing equipment information, location system, scanning devices, and CCTV surveillance. Likewise, more capacities of mobile facilities as a strategic backup will be highly useful and significant for developing countries (Singh et al. 2020; Wei 2020).

Policy guidelines encouraging the adoption of safer practices and sustainable technical solutions along with consumers’ education are the need of the hour (Parashar and Hait 2021). Moreover, restructuring policies around psychological and behavioral aspects of social awareness, incentivizing sustainable products and processes through tax cuts and discouraging low recyclable plastic products would help in achieving inclusive and sustainable plastic waste management (Patrício Silva et al. 2020, 2021; Vanapalli et al. 2021). Incorporating a decentralized waste approach and making temporary waste storage and reduction sites may be another option for waste management during unprecedented events like the COVID-10 outbreak (Kulkarni and Anantharama 2020). Overall, the most critical requirements for the efficient management of municipal solid wastes including HH wastes are; general awareness among citizens, necessary training for waste operators, reduction in single-used plastic products, and adoption of sustainable technologies for infectious wastes.

## Conclusions

Currently, the COVID-19 cases are declining and the vaccination has been started in Nepal; however, still there is a threat of pandemic and its impact in the waste management sector. Household waste as usual is a great challenge to manage and address all issues raised during pick point of outbreak and current situation. The perception of the people towards the currently existing system of household waste management is not satisfying due to various factors like weak safety and hygiene to waste collectors, lack of awareness and campaign programs from government, and disruption in collection of waste. Though there are many policies in place regarding the management of waste in Nepal, no law has been amended or formed which is suitable in the case of pandemic. Therefore, along with proper collection, segregation, and dumping of household waste and mitigation of health risks to the waste workers, who are working as the front liners, awareness to people, and effective policy formulation and implementation should be addressed for the sustainable household waste management chain amidst the pandemic.

## Data Availability

Questionnaire survey and other data can be shared upon request.

## Abbreviations

ADB: Asian development bank
ASEAN: Association of southeast Asian nations
CCTV: Closed circuit television
COVID-2019: Coronavirus disease 2019
DF: Degree of freedom
FLW: Food loss and waste
HH: Household
HHWMS: Household waste management system
ISWA: International solid waste association
LFS: Landfill site
MERS-CoV: Middle east respiratory syndrome coronavirus
MoHP: Ministry of health and population
MSW: Municipal solid waste
PCR: Polymerase chain reaction
PPE: Personal protective equipment
SARS-CoV: Severe acute respiratory syndrome coronavirus
SPSS: Statistical package for the social sciences
SWM: Solid waste management
WHO: World health organization
